# ER-Phagy and ER Stress Response (ERSR) in Plants

**DOI:** 10.3389/fpls.2019.01192

**Published:** 2019-09-27

**Authors:** Yonglun Zeng, Baiying Li, Wenxin Zhang, Liwen Jiang

**Affiliations:** ^1^School of Life Sciences, Centre for Cell and Developmental Biology and State Key Laboratory of Agrobiotechnology, The Chinese University of Hong Kong, Shatin, Hong Kong; ^2^The Chinese University of Hong Kong Shenzhen Research Institute, Shenzhen, China

**Keywords:** autophagy, ER-phagy, ER stress responses, unfolded protein response, IRE1

## Abstract

The endoplasmic reticulum (ER) is the starting point for protein secretion and lipid biosynthesis in eukaryotes. ER homeostasis is precisely regulated by the unfolded protein response (UPR) to alleviate stress, involving both transcriptional and translational regulators. Autophagy is an intracellular self-eating process mediated by the double-membrane structure autophagosome for the degradation of cytosolic components and damaged organelles to regenerate nutrient supplies under nutrient-deficient or stress conditions. A recent study has revealed that besides serving as a membrane source for phagophore formation, the ER is also tightly regulated under stress conditions by a distinct type of autophagosome, namely ER-phagy. ER-phagy has been characterized with receptors clearly identified in mammals and yeast, yet relatively little is known about plant ER-phagy and its receptors. Here, we will summarize our current knowledge of ER-phagy in yeast and mammals and highlight recent progress in plant ER-phagy studies, pointing towards a possible interplay between ER-phagy and ER homeostasis under ER stress responses (ERSRs) in plants.

## ERSR and ER-Phagy—Key Regulators for ER Homeostasis in Plant

**Figure 1:**
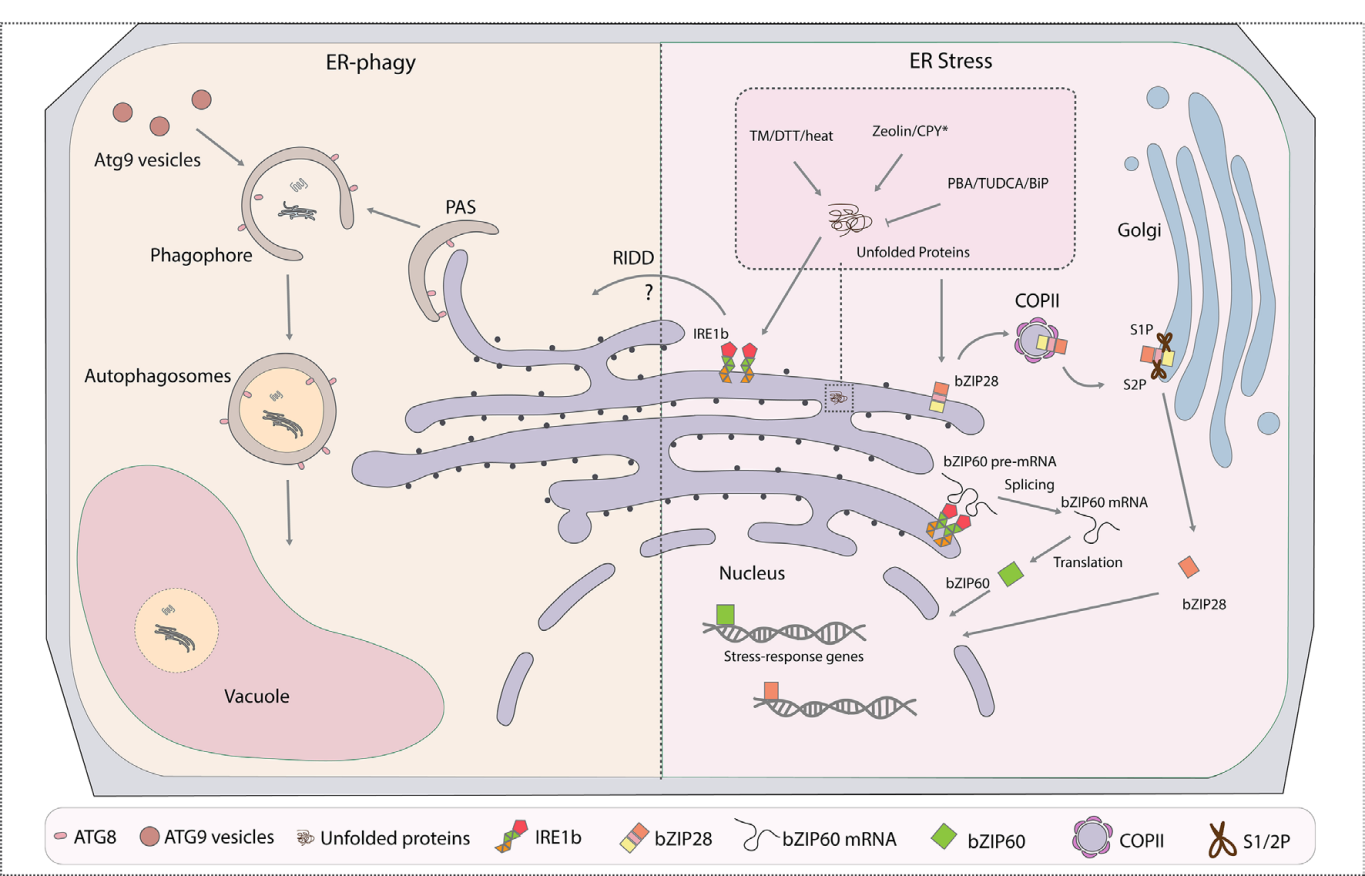
Cross talk between ER-phagy and endoplasmic reticulum (ER) stress responses (ERSRs) in plants. ER is both the major membrane source and key degradation target of autophagosomes. The part of the ER to be turned over will be engulfed at the phagophore assembly site (PAS) by forming autophagosomes through ATG8 interacting with ER-phagy receptors, which are yet to be identified in plants. The ATG9 vesicles donate essential membrane for the phagophore elongation. Mature and closed autophagosomes with cargoes enclosed in the double membrane will then fuse with the vacuole for final degradation. ERSR in plants contain two major pathways: IRE1- and bZIP28-associated pathways. In both cases, accumulation of misfolded proteins in the ER under stress conditions triggers the unfolded protein responses (UPRs). IRE1 cleaves the premature bZIP60 mRNA to achieve its mature form for the activation of downstream stress response genes under stress stimulation. On the other hand, the bZIP28 protein itself is targeted to the Golgi for cleavage and activation by proteinases S1P and S2P, and the mature form of bZIP28 reaches the nucleus for the upregulation of stress response genes. IRE1b is found to regulate both ER stress and ER-phagy responses in plants. Certain drugs that are commonly used to trigger or inhibit the UPR are highlighted in the enlarged box. PAS, phagophore assembly site; ATG, autophagic-related gene; ERSR, ER stress response; IRE1, inositol-requiring enzyme 1; bZIP, basic leucine zipper; S1P, site-1 protease; S2P, site-2 protease; TM, tunicamycin; DTT, dithiothreitol; CPY*, misfolded protein used to mimic unfolded protein accumulation; PBA, 4-phenylbutyric acid; TUDCA, tauroursodeoxycholic acid; RIDD, regulated IRE1-dependent decay; BiP, binding protein; COPII, coat protein complex II.

Genes encoding secretory proteins are translated into unfolded polypeptides by membrane-bound ribosomes and inserted into the endoplasmic reticulum (ER) lumen for proper folding, which is assisted by a set of molecular chaperones. Although the molecular machinery for protein folding in higher eukaryotes is quite elegant, protein folding is a fundamentally error-prone process. Misfolded proteins are continuously produced and monitored by the ER quality control (ERQC) system and degraded by the ER-associated degradation (ERAD) system (Ellgaard and Helenius, 2003; Braakman and Bulleid, 2011; Wan and Jiang, 2016). In higher plants, ERQC is important, as misfolded proteins can be detrimental to plant development. Nevertheless, in tissues with a high secretory activity or under adverse environmental conditions, the demands on protein folding can exceed the capacity of the ERQC and ERAD systems, causing misfolded or unfolded protein to accumulate in the ER and eventually leading to ER stress in plants. To alleviate ER stress, the unfolded protein response (UPR) is activated to refold proteins by upregulating the protein-folding machinery and degradative capacity of the ER, allowing plant development (Cao and Kaufman, 2012; Hetz, 2012; Bao et al., 2019). In yeast, the UPR is triggered upon ER stress by the ER transmembrane sensor inositol-requiring enzyme (IRE1) (Hetz and Glimcher, 2009; Hetz et al., 2011) (**Table 1**). IRE1 senses ER stress through its ER luminal sensing domain and triggers the UPR responses through splicing an mRNA encoding Hac1, the transcription factor activating the expression of ER stress response (ERSR) genes in yeast (Ron and Walter, 2007) (**Table 1**). In mammals, IRE1 is also one of the ER stress sensors, where the downstream transcription factor spliced by IRE1 is XBP1, which contains a conserved double stem-loop structure for IRE1 recognition (Yoshida et al., 2001). Autophosphorylation of IRE1 by its kinase activity may promote its interaction with other proteins such as TRAF2 in mammals (Prischi et al., 2014). Homolog to mammalian cells, the *Arabidopsis* genome encodes two IRE1 isoforms, AtIRE1a and AtIRE1b, that function as both a kinase and a ribonuclease with corresponding kinase and ribonuclease domains facing the cytosol (Howell, 2013). AtIRE1s promote the splicing of a pre-mRNA and give rise to a mature mRNA encoding bZIP60, the Hac1/XBP1 counterpart in plants, under stress stimulation (Deng et al., 2011; Nagashima et al., 2011). The spliced bZIP60 mRNA is then translated and activates the ER chaperone binding protein BiP3, triggering downstream UPRs in order to regulate ERSR (Deng et al., 2011; Nagashima et al., 2011) (**Table 1**). Plant bZIP60 does not share a high sequence similarity to Hac1 or XBP1, yet its mRNA can fold into an IRE1 recognition site in a way highly conserved from yeast to mammalians (Zhang et al., 2015). Besides IRE1, protein kinase RNA-like ER kinase (PERK) and activating transcription factor 6 (ATF6) function as other ER stress sensors to cope with the UPR in mammals (Hetz, 2012) (**Table 1**). PERK and ATF6 are both ER transmembrane proteins consisting of an ER luminal stress-sensing domain and a cytoplasmic enzymatic domain. PERK inhibits ER protein translation to relieve ER stress *via* the phosphorylation of the eukaryotic initiation factor 2 (eIF2α). On the other hand, eIF2α phosphorylation further enhances the translation of the transcription factor 4 (ATF4) to regulate UPR-related genes (Vattem and Wek, 2004). Notably, ATF4 activation is recently considered as a key signal for autophagy activation (Matsumoto et al., 2013). Distinct from IRE1 and PERK, ATF6 is transported from the ER to the Golgi apparatus through coat protein complex II (COPII) vesicles upon ER stress (Schindler and Schekman, 2009). After proteolytic cleavage in the Golgi by two proteases, site-1 protease (S1P) and S2P, the cleaved transcription factor domain of ATF6 enters the nucleus to upregulate the expression of UPR genes (Yoshida et al., 1998) (**Figure 1**). In *Arabidopsis*, PERK orthologs are yet to be identified, while two functional homologs of ATF6, bZIP28 and bZIP17, encoded in the genome have been characterized (Liu et al., 2007; Iwata and Koizumi, 2012; Kim et al., 2018; Ruberti et al., 2018). Similar to ATF6 in mammals, bZIP28 is transported from the ER to the Golgi by a sub-population of COPII vesicles for proteolytic cleavage by S1P and S2P (Liu et al., 2007; Zeng et al., 2015; Chung et al., 2016) (**Figure 1**). Although ER stress pathways for modulating ER homeostasis are evolutionarily conserved in higher eukaryotes, plants seem to utilize unique strategies to cope with differential environmental stresses, including the evolution of multi-copies of ER stress–related genes regulated by distinct biotic and abiotic stresses (Howell, 2013). Furthermore, a subset of plant-specific transcription factors, NACs [no apical meristem (NAM), *Arabidopsis* transcription activation factor (ATAF), cup-shaped cotyledon (CUC)] superfamily)], have been shown to participate in the plant UPR. Plasma membrane (PM)–localized NAC062/ANAC062/NTL6 relocates to the nucleus under stress, while inducible expression of the nucleus-localized form of NAC062 in the bZIP28 and bZIP60 double mutant (*zip28zip60*) background increases ER stress tolerance by activating the UPR genes (Yang et al., 2014a). Another membrane-anchored transcription factor, NAC089, whose expression is regulated by bZIP28 and bZIP60, also participates in ERSR by shifting from the ER to the nucleus and modulating the expression of programmed cell death (PCD)–related genes (Yang et al., 2014b). Besides membrane-anchored NAC transcription factors, the cytosolic NAC including NAC103 has also been shown to be involved in regulating the UPR gene expression (Sun et al., 2013). Mechanisms regulating the relocation of ER-localized NACs have been proved to be mainly *via* proteolytic cleavages similar to other membrane-associated transcription factors (MTFs) such as bZIP28 (Kim et al., 2007; Puranik et al., 2012). However, the underlying mechanisms regulating the relocation of NAC089 from ER to the nucleus remain elusive. NAC089 mRNA does not have the predicted double stem-loop structure, which is important for IRE1 splicing, suggesting that NAC089 might be activated in a different way from bZIP60 (Yang et al., 2014b). Further, the C-terminal ER lumen-facing tail of NAC089 is short and without a canonical S1P cutting site, which implicates that NAC089 might not be proteolytically processed in a similar manner as bZIP28 (Yang et al., 2014b). Interestingly, one rare nucleotide polymorphism caused by natural variation in the *Arabidopsis Cvi* ecotype results in premature stop and constitutive nuclear localization of NAC089, in which the C-terminus including the hydrophobic tail is not translated (Yang et al., 2014b). The existence of plant-specific NAC transcription factors functioning in stress responses further supports the notion that plants have evolved unique strategies for ERSR.

**Table 1:** ER stress regulators and their functions.

Yeast (Sc)	Mammal (Hs)	Plant (At)	Functional annotation	References
IRE1	IRE1	IRE1a/b	ER membrane–associated RNA splicing factor. In plants, IRE1b plays a major role. ER stress activates IRE1, which promotes the splicing of a pre-mRNA. IRE1 responses are delayed with respect to ATF6 responses.	Ron and Walter, 2007 Hetz and Glimcher, 2009 Liu et al., 2012 Koizumi et al., 2001
HAC1	XBP1	bZIP60	A bZIP transcription factor binds to ERSEs. IRE1 splices mRNA encoding XBP1/Hac1/bZIP60 upon ER stress activation.	Cox and Walter, 1996 Yoshida et al., 2001 Deng et al., 2011
*N.I.	ATF6	bZIP17, bZIP28	An ER type II transmembrane protein associated with the binding protein BiP/GRP78 under normal conditions but activated and trafficked to Golgi for splicing in response to ER stress.	Liu et al., 2007 Iwata and Koizumi, 2012
*N.I.	PERK	*N.I.	dsRNA-activated protein kinase-like ER kinase.	Hetz, 2012
*N.I.	eIF2α	*N.I.	Phosphorylation of the eukaryotic translation initiation factor 2 a-subunit (eIF2α) by PERK upon ER stress downregulates protein synthesis.	Vattem and Wek, 2004
*N.I.	ATF4	*N.I.	Transcriptional factor, activated by upstream PERK–eIF2α to induce ER stress apoptosis.	

Sc, Saccharomyces cerevisiae; Hs, Homo sapiens; At, Arabidopsis thaliana; IRE1, inositol-requiring enzyme; ER, endoplasmic reticulum; ATF, activating transcription factor; XBP1, X-box binding protein 1; bZIP, basic leucine zipper; ERSEs, ER stress elements; BiP, binding protein; GRP78, 78 kDa glucose regulated protein; PERK, protein kinase R-like endoplasmic reticulum kinase; eIF2α, eukaryotic translation initiation factor 2 a-subunit. *N.I., not identified.

Autophagy is an evolutionarily conserved self-eating process mediated by a double-membrane-bound organelle, the autophagosome, which encloses a portion of cytoplasm or organelles for lysosome/vacuole delivery and degradation. Autophagy-related (ATG) genes and proteins have been shown to be central to this process (Mizushima et al., 2011). The Atg proteins required for autophagosome formation consist of several functional complexes or units: the Atg1/ULK1 kinase complex, the class III phosphoinositide 3-kinase (PI3K) complex, the Atg2–Atg18 complex, the only transmembrane protein required for autophagosome biogenesis Atg9, and the two ubiquitin-like conjugation systems: the Atg12–Atg5–Atg16 axis and the Atg8 conjugation systems (Mizushima et al., 2011). The Atg12–Atg5–Atg16 system is essential for determining the site of Atg8 conjugation on forming autophagosomes where the C-terminal of pre-Atg8 is first digested by Atg4 and then transferred by the E1-like enzyme Atg7 and E2-like enzyme Atg3 to the autophagosome membranes (Mizushima et al., 2011). Due to the important role of ATG8 lipidation for autophagosome development and its stable localization on both sides of autophagosome membranes, it is used as a universal marker of autophagosomes. Plants contain not only many counterparts for yeast/mammalian Atg proteins but also some additional factors that are unique to higher plants (Li and Vierstra, 2012; Zhuang et al., 2013; Gao et al., 2015; Qi et al., 2017; Marshall and Vierstra, 2018; Zhuang et al., 2018). Although the UPR can alleviate ER stress, the overaccumulation of misfolded proteins can cause ER dysfunction and abnormal morphology. To restore ER homeostasis, selective autophagy, namely ER-phagy, is activated to degrade some of the misfolded proteins that have accumulated in the ER (Grumati et al., 2018). In yeast, ER not only provides membranes for autophagosomes but itself is a target of autophagy. Within the UPR in yeast, abnormal ER accumulating aggregated or unwanted proteins is selectively degraded by the vacuole *via* ER-phagy (Bernales et al., 2006; Schuck et al., 2009). ER-phagy requires proper cargo receptors to interact with both ATG8/LC3 and the target for degradation, so as to act as bridges between ER and the forming autophagosomes. The yeast ER-phagy pathway requires the receptors Atg39 and Atg40, two ER membrane proteins that bind Atg8 through an Atg8-interacting motif (AIM) (**Table 2**) (Mochida et al., 2015). Similarly in mammals, degradation of ER components by ER-phagy was discovered as a backup system for the inefficient proteasomal degradation of ER proteins through ERAD under the UPR. Mammalian ER-phagy receptors have been identified in recent years, including Lnp1, FAM134B, calnexin, RTN3, CCPG1, ATL3, and SEC62 (Khaminets et al., 2015; Fumagalli et al., 2016; Grumati et al., 2017; Chen et al., 2018; Smith and Wilkinson, 2018; Chen et al., 2019; Forrester et al., 2019) (**Table 2**). Evidence also emerges for the existence of plant ER-phagy in response to ER stress, where IRE1 is proved indispensable for plant ER stress–induced ER-phagy (Liu et al., 2012). In contrast, components for ER-phagy formation, function of ER-phagy upon ER stress, and ER-phagy receptors remain largely elusive in plants. Here, we summarize the progress of plant ER-phagy research, especially with respect to key regulators as well as the importance of ER-phagy to plant ER stress in the following sections.

**Table 2:** ER-phagy–associated proteins and their functions.

Organisms	Proteins	Functional annotation	References
Yeast (Sc)	Atg39	Essential for cell survival under nitrogen starvation, receptors for perinuclear ER.	Mochida et al., 2015
	Atg40	Functional counterpart of FAM134B, receptors for peripheral ER, interact with the Atg1 complex.	
Mammal (Hs)	Lnp1	Stabilizes rearrangements of the ER network.	Chen et al., 2018
	FAM134B	Reticulon-like protein present on sheet ER.	Khaminets et al., 2015
	Calnexin	Co-receptor for ER luminal misfolded procollagens Direct interaction with FAM134B.	Forrester et al., 2019
	RTN3	Tubular ER-resident protein.	Grumati et al., 2017
	SEC62	ER translocon.	Fumagalli et al., 2016
	CCPG1	A vertebrate-specific protein can interact not only with LC3/GABARAP but also with FIP200; required for efficient degradation of tubular ER.	Smith and Wilkinson, 2018
Plants (At)	IRE1b	Key regulator in ER stress responses. Nucleotide binding activity of IRE1b is required for ER stress–mediated autophagy.	Liu et al., 2012 Bao et al., 2018
	PDR2, LPR1	PDR2 is a single ER-resident P5-type ATPase (AtP5A), which controls the secretion and activity of LPR, the cell wall–targeted ferroxidase. Pi deprivation–induced ER stress–activated autophagy requires the LPR1–PDR2 module.	Naumann et al., 2019
	NAP1	A component of the SCAR/WAVE complex, required for ARP2/3-mediated actin nucleation. *nap1* mutant has reduced autophagosomes and is more sensitive to nitrogen starvation and salt stress.	Wang et al., 2016

FAM134B, family with sequence similarity 134 member B; Lnp1, lunapark1; RTN3, reticulon 3; CCPG1, cell-cycle progression gene 1; LC3, microtubule-associated protein light chain 3; GABARAP, gamma-aminobutyric acid receptor–associated protein; FIP200, 200 kDa FAK family kinase-interacting protein; PDR2, phosphate deficiency response 2; AtP5A, P5-type ATPase; LPR1, low phosphate response 1; Pi, phosphate (inorganic); NAP1, nck-associated protein 1; SCAR, suppressor of cAMP receptor; WAVE, Wiskott–Aldrich syndrome (WASP) family verprolin homologous; Arp2/3, actin-related proteins 2/3.

## IRE1b: A Bridge Between Plant Stress and Autophagy

Although increasing studies have revealed the existence of plant ER-phagy–related proteins and their potential function for ER stress, a direct link between ER-phagy and ER stress was missing until a detailed analysis of the relationship between IRE1 and autophagy was published. Since the ER is a pivotal membrane source and target of autophagy, cross talk between ER stress and ER-phagy is essential for cellular organelle and material turnover under stress conditions (Bernales et al., 2007; Hayashi-Nishino et al., 2009; Lipatova and Segev, 2015) (**Figure 1**). In mammals, the c-Jun N-terminal kinase pathway depends on the IRE1 kinase domain and is responsible for the corresponding autophagy activation. However, the components downstream of IRE1 in plants are different from those in animals, with no evidence showing the existence of parallel pathways (Urano et al., 2000; Ogata et al., 2006) (**Table 1**). Therefore, potential distinct ER stress–inducing ER-phagy mechanisms are present in plants. AtIRE1 is a key regulator in ER stress and ER-phagy responses because *ire1b* mutants result in reduced ER stress–induced autophagosome formation (Liu et al., 2012). Functional AtIRE1b, but not AtIRE1a, is required for autophagy induction by ER stress, with its target mRNA bZIP60 splicing activity not being involved (Liu et al., 2012). Previous research also revealed that autophagy can be classified based on whether its upstream signaling is dependent on ROS and NADPH oxidase (Liu et al., 2009). The NADPH oxidase inhibitor DPI is able to block nutrient deprivation and salt stress–induced autophagy but not the autophagy process under osmotic stresses (Liu et al., 2009). Both bZIP60 and bZIP28 are related to the NADPH-dependent autophagy but not the NADPH-independent IRE1b-regulated ER stress–induced autophagy, indicating that AtIRE1b is probably the sole key bridge between plant ER stress and ER-phagy responses (Liu et al., 2009). Whether the nucleotide binding and RNase activity of IRE1b connect the pathways remains to be further determined.

A very recent study elucidated another functional aspect of AtIRE1b in monitoring ER stress–induced ER-phagy through the conserved response named regulated IRE1-dependent decay (RIDD) (Bao et al., 2018). Upon acute or chronic stress in the mammalian system, IRE1 degrades ER membrane resident mRNAs *via* RIDD (Hollien et al., 2009; Chen and Brandizzi, 2013). RIDD further increases ER stress levels by degrading UPR regulators, which finally initiates apoptosis *via* suppressing antiapoptotic pre-miRNAs at the late stage of intense ER stress (Han et al., 2009; Upton et al., 2012). In the study of Bao et al. (2018), 3 out of 12 RIDD targets were found to repress autophagy upon their over-expression in plants, implying the regulatory role of AtIRE1b in ER stress–induced autophagy (Bao et al., 2018) (**Table 2**). AtIRE1b may not be the direct factor that promotes autophagy in response to ER stress but likely serves as a “licensing factor” linking ER stress to autophagy through degrading the RNA transcripts of factors that interfere with the induction of autophagy (Bao et al., 2018).

## ER-Related Autophagic Regulators and Their Potential Roles in Plant ER-Phagy

Besides IRE1b, several other ER-related regulators in plant autophagy have been recently characterized. Phosphate deficiency response 2 (PDR2) and low phosphate response 1 (LPR1) are new entries in the group of plant autophagy and ER-phagy regulators. PDR2 encodes the single P5-type ATPase of *Arabidopsis thaliana* (AtP5A), which controls the biogenesis and activity of LPR1, a cell wall–targeted ferroxidase (**Table 2**) (Ticconi et al., 2009; Muller et al., 2015; Naumann et al., 2019). PDR2 and LPR1 are reported to interact functionally and respond coordinately to iron-triggered root growth inhibition upon inorganic phosphate (Pi) limitation (Naumann et al., 2019). By comparing mutant phenotypes among autophagy, ERSRs, and local Pi deﬁciency responses, the PDR2–LPR1 module is proposed to regulate Pi deficiency–induced autophagy in root tips *via* the ER stress Ire1 axis but not through TOR-mediated macronutrient-involved systemic recycling (Naumann et al., 2019).

Nck-associated protein 1 (NAP1) is an ER-resident component of suppressor of Wiskott–Aldrich syndrome protein (WASP)/cAMP receptor (Scar)/WASP family verprolin homologous (WAVE) complex (Deeks et al., 2004). In eukaryotic cells, Arp2/3 (actin-related proteins) complex, playing an essential role in F-actin organization and cell morphogenesis, can be activated by WAVE complex *via* binding with actin filaments ((Weaver et al., 2003). *Arabidopsis* NAP1 is recruited to the double-membrane autophagic structure decorated by ATG8 upon constant pressure stress induction, where *nap1* mutants display autophagic defects, indicating the regulatory role of NAP1 in facilitating plant autophagosome biogenesis by its activating effect on actin nucleation (Wang et al., 2016). The sequential arrival of NAP1 and ATG8 to the ER membrane hints at a potential role of NAP1 in recruiting ATG proteins for forming autophagosomes neighboring to the ER. Albeit NAP1 has been demonstrated to be essential for the autophagosome biogenesis upon nitrogen starvation and high salt stress, it would be of great interest to illustrate the possible role of NAP1 in autophagosome formation upon other stresses like ER stress, as constant pressure triggers accumulation of NAP1-positive autophagosomes.

## Future Perspectives

Despite an increasing number of papers on plant ER-phagy in recent years with exciting findings, there remain open questions about the receptors and regulators that link plant stress to ER-phagy. Even though a variety of ER-phagy receptors have been characterized in yeast and mammals, plant receptors for autophagy are largely unknown. Nonetheless, plants encode homologs for most of the receptors such as Lnp1, calnexin, reticulon, ATL3, and Sec62 in its genome; whether they perform a similar function as in mammalian autophagy remains under-investigated. Besides, plant unique receptors may exist, as plants possess unique types of autophagy such as chlorophagy. Thus, future studies can be expected on the identification and characterization of plant-specific ER-phagy receptors. Furthermore, as sessile organisms, plants need to adapt to environmental changes and stresses during growth and development. Autophagy is an important biological process for nutrient recycling upon stresses. It will be exciting to resolve how plants can sense stresses such as ER stress or other biotic/abiotic stresses to activate ER-phagy to relieve plants from adverse conditions and support their growth.

## Author Contributions

YZ, BL, WZ, and LJ designed the concept and organized the manuscript. YZ, BL, and WZ wrote the manuscript. YZ and LJ edited the manuscript.

## Funding

This work was supported by grants from the Research Grants Council of Hong Kong (AoE/M-05/12, CUHK14130716, 14102417, 14100818, 14104716, C4012-16E, C4002-17G, and RIF R4005-18), the National Natural Science Foundation of China (31670179 and 91854201), and The Chinese University of Hong Kong (CUHK) Research Committee to LJ.

## Conflict of Interest

The authors declare that the research was conducted in the absence of any commercial or financial relationships that could be construed as a potential conflict of interest

## References

[B1] Bao Y. Bassham D. C. Howell S. H. (2019). A functional unfolded protein response is required for normal vegetative development. Plant Physiol. 179, 1834–1843. 10.1104/pp.18.01261 30710050 PMC6446744

[B2] Bao Y. Pu Y. Yu X. Gregory B. D. Srivastava R. Howell S. H. (2018). IRE1B degrades RNAs encoding proteins that interfere with the induction of autophagy by ER stress in *Arabidopsis thaliana*. Autophagy 14, 1562–1573. 10.1080/15548627.2018.1462426 29940799 PMC6135571

[B3] Bernales S. Papa F. R. Walter P. (2006). Intracellular signaling by the unfolded protein response. Annu. Rev. Cell Dev. Biol. 22, 487–508. 10.1146/annurev.cellbio.21.122303.120200 16822172

[B4] Bernales S. Schuck S. Walter P. (2007). ER-phagy: selective autophagy of the endoplasmic reticulum. Autophagy 3, 285–287. 10.4161/auto.3930 17351330

[B5] Braakman I. Bulleid N. J. (2011). Protein folding and modification in the mammalian endoplasmic reticulum. Annu. Rev. Biochem. 80, 71–99. 10.1146/annurev-biochem-062209-093836 21495850

[B6] Cao S. S. Kaufman R. J. (2012). Unfolded protein response. Curr. Biol. 22, R622–R626. 10.1016/j.cub.2012.07.004 22917505

[B7] Chen Q. Xiao Y. Chai P. Zheng P. Teng J. Chen J. (2019). ATL3 is a tubular ER-phagy receptor for GABARAP-mediated selective autophagy. Curr. Biol. 29, 846–855 e846. 10.1016/j.cub.2019.01.041 30773365

[B8] Chen S. Cui Y. Parashar S. Novick P. J. Ferro-Novick S. (2018). ER-phagy requires Lnp1, a protein that stabilizes rearrangements of the ER network. Proc. Natl. Acad. Sci. U. S. A. 115, E6237–E6244. 10.1073/pnas.1805032115 29915089 PMC6142256

[B9] Chen Y. Brandizzi F. (2013). IRE1: ER stress sensor and cell fate executor. Trends Cell Biol. 23, 547–555. 10.1016/j.tcb.2013.06.005 23880584 PMC3818365

[B10] Chung K. P. Zeng Y. Jiang L. (2016). COPII paralogs in plants: functional redundancy or diversity? Trends Plant Sci. 21, 758–769. 10.1016/j.tplants.2016.05.010 27317568

[B11] Cox J. S. Walter P. (1996). A novel mechanism for regulating activity of a transcription factor that controls the unfolded protein response. Cell 87, 391–404. 10.1016/S0092-8674(00)81360-4 8898193

[B12] Deeks M. J. Kaloriti D. Davies B. Malho R. Hussey P. J. (2004). *Arabidopsis* NAP1 is essential for Arp2/3-dependent trichome morphogenesis. Curr. Biol. 14, 1410–1414. 10.1016/j.cub.2004.06.065 15296761

[B13] Deng Y. Humbert S. Liu J. X. Srivastava R. Rothstein S. J. Howell S. H. (2011). Heat induces the splicing by IRE1 of a mRNA encoding a transcription factor involved in the unfolded protein response in *Arabidopsis*. Proc. Natl. Acad. Sci. U. S. A. 108, 7247–7252. 10.1073/pnas.1102117108 21482766 PMC3084119

[B14] Ellgaard L. Helenius A. (2003). Quality control in the endoplasmic reticulum. Nat. Rev. Mol. Cell Biol. 4, 181–191. 10.1038/nrm1052 12612637

[B15] Forrester A. De Leonibus C. Grumati P. Fasana E. Piemontese M. Staiano L. (2019). A selective ER-phagy exerts procollagen quality control via a Calnexin-FAM134B complex. EMBO J. 38:e99847. 10.15252/embj.201899847 30559329 PMC6331724

[B16] Fumagalli F. Noack J. Bergmann T. J. Cebollero E. Pisoni G. B. Fasana E. (2016). Translocon component Sec62 acts in endoplasmic reticulum turnover during stress recovery. Nat. Cell Biol. 18, 1173–1184. 10.1038/ncb3423 27749824

[B17] Gao C. Zhuang X. Cui Y. Fu X. He Y. Zhao Q. (2015). Dual roles of an *Arabidopsis* ESCRT component FREE1 in regulating vacuolar protein transport and autophagic degradation. Proc. Natl. Acad. Sci. U. S. A. 112, 1886–1891. 10.1073/pnas.1421271112 25624505 PMC4330779

[B18] Grumati P. Dikic I. Stolz A. (2018). ER-phagy at a glance. J. Cell Sci. 131, jcs217364. 10.1242/jcs.217364 30177506

[B19] Grumati P. Morozzi G. Holper S. Mari M. Harwardt M. I. Yan R. (2017). 674 Full length RTN3 regulates turnover of tubular endoplasmic reticulum via 675 selective autophagy. Elife 6:e25555. 10.7554/eLife.25555 28617241 PMC5517149

[B20] Han D. Lerner A. G. Vande Walle L. Upton J. P. Xu W. Hagen A. (2009). IRE1alpha kinase activation modes control alternate endoribonuclease outputs to determine divergent cell fates. Cell 138, 562–575. 10.1016/j.cell.2009.07.017 19665977 PMC2762408

[B21] Hayashi-Nishino M. Fujita N. Noda T. Yamaguchi A. Yoshimori T. Yamamoto A. (2009). A subdomain of the endoplasmic reticulum forms a cradle for autophagosome formation. Nat. Cell Biol. 11, 1433–1437. 10.1038/ncb1991 19898463

[B22] Hetz C. (2012). The unfolded protein response: controlling cell fate decisions under ER stress and beyond. Nat. Rev. Mol. Cell Biol. 13, 89–102. 10.1038/nrm3270 22251901

[B23] Hetz C. Glimcher L. H. (2009). Fine-tuning of the unfolded protein response: assembling the IRE1alpha interactome. Mol. Cell 35, 551–561. 10.1016/j.molcel.2009.08.021 19748352 PMC3101568

[B24] Hetz C. Martinon F. Rodriguez D. Glimcher L. H. (2011). The unfolded protein response: integrating stress signals through the stress sensor IRE1 alpha. Physiol. Rev. 91, 1219–1243. 10.1152/physrev.00001.2011 22013210

[B25] Hollien J. Lin J. H. Li H. Stevens N. Walter P. Weissman J. S. (2009). Regulated Ire1-dependent decay of messenger RNAs in mammalian cells. J. Cell Biol. 186, 323–331. 10.1083/jcb.200903014 19651891 PMC2728407

[B26] Howell S. H. (2013). Endoplasmic reticulum stress responses in plants. Annu. Rev. Plant Biol. 64, 477–499. 10.1146/annurev-arplant-050312-120053 23330794

[B27] Iwata Y. Koizumi N. (2012). Plant transducers of the endoplasmic reticulum unfolded protein response. Trends Plant Sci. 17, 720–727. 10.1016/j.tplants.2012.06.014 22796463

[B28] Khaminets A. Heinrich T. Mari M. Grumati P. Huebner A. K. Akutsu M. (2015). Regulation of endoplasmic reticulum turnover by selective autophagy. Nature 522, 354–358. 10.1038/nature14498 26040720

[B29] Kim J. S. Yamaguchi-Shinozaki K. Shinozaki K. (2018). ER-anchored transcription factors bZIP17 and bZIP28 regulate root elongation. Plant Physiol. 176, 2221–2230. 10.1104/pp.17.01414 29367234 PMC5841724

[B30] Kim S. Y. Kim S. G. Kim Y. S. Seo P. J. Bae M. Yoon H. K. (2007). Exploring membrane-associated NAC transcription factors in *Arabidopsis*: implications for membrane biology in genome regulation. Nucleic Acids Res. 35, 203–213. 10.1093/nar/gkl1068 17158162 PMC1802569

[B31] Koizumi N. Martinez I.M. Kimata Y. Kohno K. Sano H. Chrispeels M.J. (2001). Molecular characterization of two Arabidopsis Ire1 homologs, endoplasmic reticulum-located transmembrane protein kinases. Plant Physiol 127, 949–962. 11706177 PMC129266

[B32] Li F. Vierstra R. D. (2012). Autophagy: a multifaceted intracellular system for bulk and selective recycling. Trends Plant Sci. 17, 526–537. 10.1016/j.tplants.2012.05.006 22694835

[B33] Lipatova Z. Segev N. (2015). A role for macro-ER-phagy in ER quality control. PLoS Genet. 11, e1005390. 10.1371/journal.pgen.1005390 26181331 PMC4504476

[B34] Liu J. X. Srivastava R. Che P. Howell S. H. (2007). An endoplasmic reticulum stress response in *Arabidopsis* is mediated by proteolytic processing and nuclear relocation of a membrane-associated transcription factor, bZIP28. Plant Cell 19, 4111–4119. 10.1105/tpc.106.050021 18156219 PMC2217655

[B35] Liu Y. Burgos J. S. Deng Y. Srivastava R. Howell S. H. Bassham D. C. (2012). Degradation of the endoplasmic reticulum by autophagy during endoplasmic reticulum stress in *Arabidopsis*. Plant Cell 24, 4635–4651. 10.1105/tpc.112.101535 23175745 PMC3531857

[B36] Liu Y. Xiong Y. Bassham D. C. (2009). Autophagy is required for tolerance of drought and salt stress in plants. Autophagy 5, 954–963. 10.4161/auto.5.7.9290 19587533

[B37] Marshall R. S. Vierstra R. D. (2018). Autophagy: the master of bulk and selective recycling. Annu. Rev. Plant Biol. 69, 173–208. 10.1146/annurev-arplant-042817-040606 29539270

[B38] Matsumoto H. Miyazaki S. Matsuyama S. Takeda M. Kawano M. Nakagawa H. (2013). Selection of autophagy or apoptosis in cells exposed to ER-stress depends on ATF4 expression pattern with or without CHOP expression. Biol. Open 2, 1084–1090. 10.1242/bio.20135033 24167719 PMC3798192

[B39] Mizushima N. Yoshimori T. Ohsumi Y. (2011). The role of Atg proteins in autophagosome formation. Annu. Rev. Cell Dev. Biol. 27, 107–132. 10.1146/annurev-cellbio-092910-154005 21801009

[B40] Mochida K. Oikawa Y. Kimura Y. Kirisako H. Hirano H. Ohsumi Y. (2015). Receptor-mediated selective autophagy degrades the endoplasmic reticulum and the nucleus. Nature 522, 359–362. 10.1038/nature14506 26040717

[B41] Muller J. Toev T. Heisters M. Teller J. Moore K. L. Hause G. (2015). Iron-dependent callose deposition adjusts root meristem maintenance to phosphate availability. Dev. Cell 33, 216–230. 10.1016/j.devcel.2015.02.007 25898169

[B42] Nagashima Y. Mishiba K. Suzuki E. Shimada Y. Iwata Y. Koizumi N. (2011). *Arabidopsis* IRE1 catalyses unconventional splicing of bZIP60 mRNA to produce the active transcription factor. Sci. Rep. 1, 29. 10.1038/srep00029 22355548 PMC3216516

[B43] Naumann C. Muller J. Sakhonwasee S. Wieghaus A. Hause G. Heisters M. (2019). The local phosphate deficiency response activates endoplasmic reticulum stress–dependent autophagy. Plant Physiol. 179, 460–476. 10.1104/pp.18.01379 30510038 PMC6426416

[B44] Ogata M. Hino S. Saito A. Morikawa K. Kondo S. Kanemoto S. (2006). Autophagy is activated for cell survival after endoplasmic reticulum stress. Mol. Cell Biol. 26, 9220–9231. 10.1128/MCB.01453-06 17030611 PMC1698520

[B45] Prischi F. Nowak P. R. Carrara M. Ali M. M. (2014). Phosphoregulation of Ire1 RNase splicing activity. Nat. Commun. 5, 3554. 10.1038/ncomms4554 24704861 PMC3988810

[B46] Puranik S. Sahu P. P. Srivastava P. S. Prasad M. (2012). NAC proteins: regulation and role in stress tolerance. Trends Plant Sci. 17, 369–381. 10.1016/j.tplants.2012.02.004 22445067

[B47] Qi H. Xia F. N. Xie L. J. Yu L. J. Chen Q. F. Zhuang X. H. (2017). TRAF family proteins regulate autophagy dynamics by modulating AUTOPHAGY PROTEIN6 stability in *Arabidopsis*. Plant Cell 29, 890–911. 10.1105/tpc.17.00056 28351989 PMC5435438

[B48] Ron D. Walter P. (2007). Signal integration in the endoplasmic reticulum unfolded protein response. Nat. Rev. Mol. Cell Biol. 8, 519–529. 10.1038/nrm2199 17565364

[B49] Ruberti C. Lai Y. Brandizzi F. (2018). Recovery from temporary endoplasmic reticulum stress in plants relies on the tissue-specific and largely independent roles of bZIP28 and bZIP60, as well as an antagonizing function of BAX-Inhibitor 1 upon the pro-adaptive signaling mediated by bZIP28. Plant J. 93, 155–165. 10.1111/tpj.13768 29124827 PMC5732024

[B50] Schindler A. J. Schekman R. (2009). In vitro reconstitution of ER-stress induced ATF6 transport in COPII vesicles. Proc. Natl. Acad. Sci. U. S. A. 106, 17775–17780. 10.1073/pnas.0910342106 19822759 PMC2764917

[B51] Schuck S. Prinz W. A. Thorn K. S. Voss C. Walter P. (2009). Membrane expansion alleviates endoplasmic reticulum stress independently of the unfolded protein response. J. Cell Biol. 187, 525–536. 10.1083/jcb.200907074 19948500 PMC2779237

[B52] Smith M. D. Wilkinson S. (2018). CCPG1, a cargo receptor required for reticulophagy and endoplasmic reticulum proteostasis. Autophagy 14, 1090–1091. 10.1080/15548627.2018.1441473 29916296 PMC6103402

[B53] Sun L. Yang Z. T. Song Z. T. Wang M. J. Sun L. Lu S. J. (2013). The plant-specific transcription factor gene NAC103 is induced by bZIP60 through a new cis-regulatory element to modulate the unfolded protein response in *Arabidopsis*. Plant J. 76, 274–286. 10.1111/tpj.12287 23869562

[B54] Ticconi C. A. Lucero R. D. Sakhonwasee S. Adamson A. W. Creff A. Nussaume L. (2009). ER-resident proteins PDR2 and LPR1 mediate the developmental response of root meristems to phosphate availability. Proc. Natl. Acad. Sci. U. S. A. 106, 14174–14179. 10.1073/pnas.0901778106 19666499 PMC2723163

[B55] Upton J. P. Wang L. Han D. Wang E. S. Huskey N. E. Lim L. (2012). IRE1alpha cleaves select microRNAs during ER stress to derepress translation of proapoptotic Caspase-2. Science 338, 818–822. 10.1126/science.1226191 23042294 PMC3742121

[B56] Urano F. Wang X. Bertolotti A. Zhang Y. Chung P. Harding H. P. (2000). Coupling of stress in the ER to activation of JNK protein kinases by transmembrane protein kinase IRE1. Science 287, 664–666. 10.1126/science.287.5453.664 10650002

[B57] Vattem K. M. Wek R. C. (2004). Reinitiation involving upstream ORFs regulates ATF4 mRNA translation in mammalian cells. Proc. Natl. Acad. Sci. U. S. A. 101, 11269–11274. 10.1073/pnas.0400541101 15277680 PMC509193

[B58] Wan S. Jiang L. (2016). Endoplasmic reticulum (ER) stress and the unfolded protein response (UPR) in plants. Protoplasma 253, 753–764. 10.1007/s00709-015-0842-1 26060134

[B59] Wang P. Richardson C. Hawes C. Hussey P. J. (2016). *Arabidopsis* NAP1 regulates the formation of autophagosomes. Curr. Biol. 26, 2060–2069. 10.1016/j.cub.2016.06.008 27451899

[B60] Weaver A. M. Young M. E. Lee W. L. Cooper J. A. (2003). Integration of signals to the Arp2/3 complex. Curr. Opin. Cell Biol. 15, 23–30. 10.1016/S0955-0674(02)00015-7 12517700

[B61] Yang Z. T. Lu S. J. Wang M. J. Bi D. L. Sun L. Zhou S. F. (2014a). A plasma membrane–tethered transcription factor, NAC062/ANAC062/NTL6, mediates the unfolded protein response in *Arabidopsis*. Plant J. 79, 1033–1043. 10.1111/tpj.12604 24961665

[B62] Yang Z. T. Wang M. J. Sun L. Lu S. J. Bi D. L. Sun L. (2014b). The membrane-associated transcription factor NAC089 controls ER-stress–induced programmed cell death in plants. PLoS Genet. 10, e1004243. 10.1371/journal.pgen.1004243 24675811 PMC3967986

[B63] Yoshida H. Haze K. Yanagi H. Yura T. Mori K. (1998). Identification of the cis-acting endoplasmic reticulum stress response element responsible for transcriptional induction of mammalian glucose-regulated proteins. Involvement of basic leucine zipper transcription factors. J. Biol. Chem. 273, 33741–33749. 10.1074/jbc.273.50.33741 9837962

[B64] Yoshida H. Matsui T. Yamamoto A. Okada T. Mori K. (2001). XBP1 mRNA is induced by ATF6 and spliced by IRE1 in response to ER stress to produce a highly active transcription factor. Cell 107, 881–891. 10.1016/S0092-8674(01)00611-0 11779464

[B65] Zeng Y. Chung K. P. Li B. Lai C. M. Lam S. K. Wang X. (2015). Unique COPII component AtSar1a/AtSec23a pair is required for the distinct function of protein ER export in *Arabidopsis thaliana*. Proc. Natl. Acad. Sci. U. S. A. 112, 14360–14365. 10.1073/pnas.1519333112 26578783 PMC4655569

[B66] Zhang L. Chen H. Brandizzi F. Verchot J. Wang A. (2015). The UPR branch IRE1–bZIP60 in plants plays an essential role in viral infection and is complementary to the only UPR pathway in yeast. PLoS Genet. 11, e1005164. 10.1371/journal.pgen.1005164 25875739 PMC4398384

[B67] Zhuang X. Chung K. P. Luo M. Jiang L. (2018). Autophagosome biogenesis and the endoplasmic reticulum: a plant perspective. Trends Plant Sci. 23, 677–692. 10.1016/j.tplants.2018.05.002 29929776

[B68] Zhuang X. Wang H. Lam S. K. Gao C. Wang X. Cai Y. (2013). A BAR-domain protein SH3P2, which binds to phosphatidylinositol 3-phosphate and ATG8, regulates autophagosome formation in *Arabidopsis*. Plant Cell 25, 4596–4615 10.1105/tpc.113.118307 24249832 PMC3875738

